# Best Practices for Teaching Psychotherapy to Medical Students: A Scoping Review

**DOI:** 10.3390/bs15060780

**Published:** 2025-06-05

**Authors:** Marie-Hélène Garon, Geneviève Létourneau, David Caron, Léa Renaud-Cloutier, Marie Désilets, Alexandre Hudon

**Affiliations:** 1 Department of Psychiatry and Addictology, Faculty of Medicine, Université de Montréal, Montreal, QC H3T 1J4, Canada; marie-helene.garon@umontreal.ca (M.-H.G.); genevieve.letourneau@umontreal.ca (G.L.); david.caron.3@umontreal.ca (D.C.); lea.renaud-cloutier@umontreal.ca (L.R.-C.); 2 Department of Psychiatry, Institut Universitaire de Gériatrie de Montréal, Montreal, QC H3W 1W5, Canada; 3 Department of Psychiatry, Institut Universitaire en Santé Mentale de Montréal, Montreal, QC H1N 3M5, Canada; mdesilets.iusmm@ssss.gouv.qc.ca; 4 Centre de Recherche de l’Institut Universitaire en Santé Mentale de Montréal, Montreal, QC H1N 3V2, Canada; 5 Institut Nationale de Psychiatrie légale Philippe-Pinel, Montreal, QC H1C 1H1, Canada; 6 Groupe Interdisciplinaire de Recherche sur la Cognition et le Raisonnement Professionel (GIRCoPRo), Université de Montréal, Montreal, QC H3C 3J7, Canada

**Keywords:** psychotherapy education, medical students, medical education, motivational interviewing, role play, clinical exposition, e-learning, curriculum development, experiential learning, psychiatry teaching

## Abstract

Psychotherapy is an essential component of mental healthcare, yet its formal instruction within medical curricula remains underdeveloped. This scoping review aimed to map the best practices for teaching psychotherapy to medical students by examining the types of psychotherapy covered and the teaching strategies employed. A systematic search was conducted across the PubMed, Embase, PsycINFO and Google Scholar databases without time restrictions, and studies were selected if they focused on psychotherapy education for medical students. Fifteen studies met the inclusion criteria. The findings revealed that multimodal approaches, combining didactic sessions, experiential learning, clinical exposure and digital content, were the most commonly used and pedagogically effective strategies. Role play and clinical exposition were particularly valued for enhancing communication skills, empathy and therapeutic understanding, while e-learning emerged as a flexible but less frequently used tool. Motivational interviewing was the most frequently taught psychotherapeutic modality, followed by mindfulness, cognitive-behavioral therapy and psychodynamic approaches. Although the overall quality of studies was moderate to high, the heterogeneity in study design and outcome measures limited direct comparisons. These results highlight the need for standardized, experiential and integrated teaching strategies to better prepare future physicians for incorporating psychotherapy principles into clinical practice.

## 1. Introduction

### 1.1. Psychotherapy

Psychotherapy is defined as a modality of treatment in which the therapist and patient work together to ameliorate psychopathologic conditions and functional impairment through focus on the therapeutic relationship and the patient’s attitudes, thoughts, affect and behavior, as well as social context and development (Brent & Kolko, 1998). Research consistently supports psychotherapy as being an effective and cost-efficient intervention for numerous psychological, behavioral and somatic conditions (Cook et al., 2017). Despite the common use of medication, addressing psychosocial factors is often essential for optimal treatment outcomes (Craighead & Craighead, 2001). Numerous meta-analyses demonstrate psychotherapy’s efficacy, sometimes yielding comparable or superior results to pharmacological treatments (Leichsenring et al., 2022; Kamenov et al., 2017). In fact, the growing body of literature highlights the importance of integrating psychotherapy into broader treatment plans to ensure long-term patient well-being and to prevent relapse in chronic mental health conditions. The development of evidence-based psychotherapeutic methods can be traced back to the early 20th century and various approaches have since been widely adopted (Marks, 2017). Over time, three major streams of psychotherapy have emerged, offering distinct perspectives on human experiences and mental health (Karasu, 1977). The first approach originates from Freud’s pioneering work, which presented a compelling framework underscoring the unconscious mind’s powerful influence on daily life (Bargh & Morsella, 2008). The second laid the foundation for the cognitive–behavioral movement, grounded in empirical observations of human behavior. The third focuses on humanistic approaches, highlighting self-determination and phenomenological perspectives in therapeutic practice (Solobutina & Miyassarova, 2019). Over the years, these methods have evolved to incorporate advancements in neuroscience, cognitive science and digital health interventions, further expanding their applicability. Psychotherapy has proven beneficial across diverse populations, regardless of age, education, ethnicity or cultural background (Kennedy et al., 2016).

### 1.2. Medical Education

In North America, a Doctor of Medicine (MD) degree typically takes between three and five years to complete and is usually divided into a pre-clinical phase (pre-clerkship) and a clinical phase (clerkship) (Boivin & Sakurai, 2024; McOwen et al., 2020). As an example, the University of British Columbia has a 4-year competency-based curriculum, including two years of pre-clinical training and two years of clinical training, where students rotate through various medical specialties in hospital and community settings. Similarly, at the Université de Montréal, the first-cycle doctorate in medicine is a 200-credit program consisting of a preparatory year, two years of pre-clinical training and two years of clinical training. In this case, psychiatry is covered in the pre-clinical phase through a five-week learning block that includes large-group lectures and small-group problem-based learning sessions. In the clinical phase, students complete a mandatory six-week psychiatry rotation and have the option to undertake additional psychiatry electives, ranging in duration from zero to eight weeks. Elective opportunities in psychiatry are widely available in North American medical schools, allowing students to explore various aspects of the field, including psychotherapy. However, these electives remain optional and many students choose not to pursue them, as the majority aim for a career in general practice.

### 1.3. Psychotherapy in Medical Education

Despite the global burden of mental illness and the recognized importance of psychosocial interventions, psychotherapy training is inconsistently integrated into undergraduate medical education worldwide. For example, while some North American and European programs offer brief exposure through electives, others (particularly in low- and middle-income countries) report minimal or no formal instruction, reinforcing a biomedical orientation and limiting future clinicians’ psychotherapeutic competencies. As another example, in North America, the practice of psychotherapy is regulated differently across U.S. states and Canadian provinces and territories. While medical graduates can usually practice psychotherapy after meeting the necessary educational and regulatory requirements, these requirements differ by jurisdiction. As an example, in the Canadian province of Québec, a permit from the *Ordre des psychologues du Québec* is required to practice psychotherapy, whether as a psychologist, physician, or licensed psychotherapist. In fact, all healthcare professionals, other than physicians and psychologists, seeking a psychotherapy permit must hold a master’s degree in the field of mental health and human relations and must complete 765 h of university-level theoretical training and 600 h of supervised internship specifically in psychotherapy. In this province, every medical graduate automatically obtains the right to practice psychotherapy, even though there is a significant gap between the competencies required to practice psychotherapy and the general medical curriculum. Indeed, in medical schools, psychiatry and psychological health represent only a small fraction of overall medical education (Stoudemire, 1996). Most medical students receive minimal specific training on psychotherapy, while other programs, such as psychology, are entirely dedicated to teaching and applying it (Daniel et al., 1990). As a result, many medical students perceive psychotherapy as being irrelevant to their future role as general practitioners, believing it is not a skill they can realistically develop. Therefore, many newly trained physicians may feel unprepared to integrate psychotherapeutic techniques into their practice, potentially leading to an over-reliance on pharmacological interventions when managing mental health disorders. Improving psychotherapy training in medical education is crucial to better equip future physicians with the necessary tools to address the needs of their patients, especially given that mental health concerns account for a significant proportion of consultations with family physicians. By enhancing their ability to incorporate psychotherapeutic techniques into their practice, physicians can provide more comprehensive, patient-centered care, improving the overall management of mental health.

### 1.4. Objectives of This Scoping Review

The objective of this scoping review is to identify the best practices in teaching psychotherapy to medical students. Specifically, it aims to determine which types of psychotherapy are covered in medical education and to explore the various teaching and learning methods used to enhance students’ psychotherapy-related skills. By mapping the existing literature on this topic, this review seeks to provide insights into effective educational approaches and contribute to the improvement in psychotherapy training in medical curricula. It is anticipated that psychotherapy training in medical schools primarily focuses on supportive therapy or motivational interviewing, with less emphasis being placed on other therapeutic models. We also hypothesize that the most effective teaching methods will likely combine structured theoretical learning with practical experiences such as role-playing or clinical simulations.

## 2. Materials and Methods

### 2.1. Search Strategies

A comprehensive search was conducted across the electronic databases PubMed, Embase, PsycINFO and Google Scholar from their inception to 2024. The search strategy combined medical subject headings (MeSHs) and free-text keywords related to medical students (e.g., students, medical, clinical clerkship, internships and clerkships), psychotherapy (e.g., psychotherapy, cognitive–behavioral therapy, psychodynamic, transference and defense mechanisms) and education (e.g., teaching, learning, education, medical, curriculum and training). The strategy aimed to capture a broad range of studies on how psychotherapy is taught in medical education. The complete electronic search strategy is provided in Supplementary Material S1. The search methodology was collaboratively developed by MD, an academic librarian with expertise in health sciences education. Searches were performed by MHG and were independently verified by AH in September 2024. No date, setting or geographic restrictions were applied, but only articles published in English or French were included. The PRISMA for Scoping Reviews (PRISMA-ScR) was followed for this study and is reported in Supplementary Material S2. This study was not pre-registered.

### 2.2. Study Eligibility

Studies were eligible for inclusion if they met the following criteria: (1) the study focuses on the field of psychotherapy; (2) it focused on educational or learning strategies related to psychotherapy; (3) the population under study included medical students at any stage of their training; and (4) the article was published in English or French. Studies were excluded if they were not formally published in peer-reviewed journals (e.g., preprints), or if they were opinion pieces, book chapters or letters to the Editor. The inclusion criteria were designed to identify the peer-reviewed literature that explores concrete approaches to teaching psychotherapy within medical education, while excluding non-empirical or informal commentary.

### 2.3. Data Extraction

Data extraction was performed using a standardized charting table developed by the research team in Microsoft Excel (version 17.0). The following data were extracted from each included study: authors, year of publication, title, population studied (e.g., level or type of medical students), type of psychotherapy taught, teaching method used and main findings or outcomes. This approach allowed for the consistent documentation of the identified studies’ characteristics and facilitated the identification of trends across different educational strategies and psychotherapeutic approaches. Data were extracted by MHG and LRC and were verified by GL and AH to ensure accuracy and completeness.

### 2.4. Quality Assessment

To enhance the interpretability of the findings, a structured quality assessment of the included studies was performed using two validated tools—the Best Evidence in Medical Education (BEME) checklist and the Medical Education Research Study Quality Instrument (MERSQI) (Harden et al., 1999; Jaros & Beck Dallaghan, 2024). The BEME checklist is a broadly applicable tool that is designed to assess the methodological quality of medical education studies, regardless of study design. It consists of 11 criteria evaluating the clarity of research questions, the appropriateness of methods, context description, outcome measures and the coherence of conclusions, with each item being scored as “Yes” (1), “Partial” (0.5) or “No” (0), for a maximum score of 11. Studies scoring ≥9 were considered high quality, studies scoring 7–8.5 were considered moderate quality and studies scoring <7 were considered low quality. In addition, the MERSQI was used for studies employing quantitative methods. This instrument evaluates six domains—study design, sampling, type of data, validity of evaluation instruments, data analysis and outcome level. Each domain is scored from 0 to 3, for a total score ranging from 5 to 18. Together, these tools provided a structured evaluation of methodological rigor across a diverse set of educational studies.

## 3. Results

### 3.1. Description of Studies

**Figure 1:**
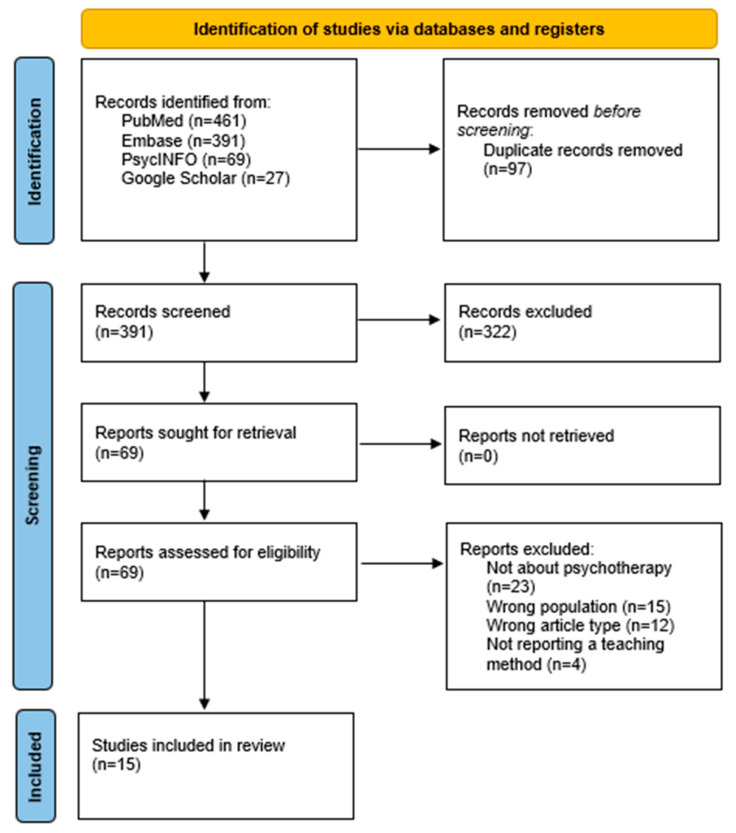
PRISMA (Preferred Reporting Items for Systematic Reviews and Meta-Analyses) flowchart for the inclusion of studies.

The present scoping review explored the best practices for teaching psychotherapy to medical students. An initial search across the four databases yielded 948 records. After the removal of 97 duplicate entries, 391 records were screened based on their titles and abstracts. From this initial screening, 322 records were excluded for not meeting basic inclusion criteria. A total of 69 full-text articles were then assessed in detail for eligibility. Of these, 54 were excluded for the following reasons: the article was not focused on psychotherapy (n = 23), involved the wrong population (n = 15), was of an ineligible article type such as letters or book chapters (n = 12) or did not describe a teaching method (n = 4). This screening process resulted in 15 studies being included in the final review. A detailed flowchart of the selection process is presented in Figure 1, and a full list of included studies can be found in Table 1.

### 3.2. Main Results

Among the 15 articles included in this scoping review, e-learning was identified in 4 studies, which highlighted its value as a flexible and scalable tool for delivering foundational psychotherapy knowledge. Role play was featured in nine studies, making it one of the most widely used methods; it was praised for fostering communication skills, empathy and self-reflection in a safe and engaging environment. Clinical exposition, involving direct patient interaction or observation, was also present in nine studies and was recognized for its ability to contextualize psychotherapeutic concepts and reinforce professional identity through experiential learning. Finally, multimodal approaches were the most frequently used, with 13 studies combining various techniques (such as lectures, role play, clinical exposure and digital content) to engage cognitive, emotional and behavioral domains. This layered approach was consistently associated with enhanced learner satisfaction, deeper understanding and greater readiness for clinical practice.

Across the included studies, motivational interviewing emerged as the most commonly taught psychotherapeutic approach (n = 7), valued for its brevity, structure and applicability in various clinical contexts. Motivational interview training consistently led to measurable improvements in students’ communication skills, empathy and confidence in patient-centered interactions. Mindfulness-based interventions (n = 2) and cognitive–behavioral therapy (n = 1) were also frequently integrated, often through brief workshops or structured modules, enhancing self-awareness, stress regulation and therapeutic engagement. Psychodynamic principles (n = 1), though less frequently addressed, were associated with a deeper understanding of the doctor–patient relationship and reflective practice. Four of the identified studies related to multiple therapeutic approaches.

#### 3.2.1. E-Learning

E-learning was featured in a few studies as an accessible and scalable method for delivering foundational psychotherapy knowledge. Truong et al. reported on a randomized trial comparing computer-guided solo instruction with traditional teaching in exposure therapy, showing knowledge gains despite methodological limitations (Truong et al., 2015). Keifenheim et al. evaluated a blended-learning approach combining video modules with in-person skill sessions, which led to improved competence in motivational interviewing (Keifenheim et al., 2019). Edwards explored the integration of online materials with asynchronous discussion boards in an interprofessional training model, reporting positive engagement from learners (Edwards et al., 2022). Similarly, Katlman embedded digital tools within a psychiatric interviewing curriculum, finding increased student confidence in approaching sensitive topics (Kaltman et al., 2015). These studies demonstrate that e-learning, especially when paired with interactive elements, can be a useful adjunct for psychotherapy education.

#### 3.2.2. Role Play

Role play was one of the most commonly used and effective teaching methods across the reviewed studies. King et al. introduced role lay-based learning (RBL) in psychiatry, where students alternated the roles of clinician and patient, leading to improvements in empathy and preparedness for clinical exams (King et al., 2015). Selzer et al. emphasized demystifying psychotherapy through metaphors and hands-on role play exercises (Selzer et al., 2015). Neufeld et al. and Muzyk et al. used structured role plays as part of SBIRT and motivational interviewing training, respectively, allowing students to simulate clinical encounters (Neufeld et al., 2012; Muzyk et al., 2019). Chéret and Buck also implemented role play within workshops on psychotherapeutic interviewing, highlighting its ability to build confidence (Chéret et al., 2018; Buck et al., 2021). McKenzie et al. indirectly evaluated the impact of experiential exposure (including role play) on students’ attitudes toward mindfulness as an intervention (McKenzie et al., 2012). Merlo et al. integrated role playing in cognitive restructuring sessions, with strong student satisfaction (Merlo et al., 2022). Truong et al. further confirmed that role play is commonly included in the most effective interventions (Truong et al., 2015). Overall, role play offers a safe space for students to develop psychotherapeutic communication and self-reflective skills.

#### 3.2.3. Clinical Exposition

Clinical exposition (a direct interaction with or observation of patients) was highlighted as a key component in deepening students’ understanding of psychotherapy. Mintz strongly advocated for the reintroduction of psychoanalytic concepts into medical curricula through patient-centered approaches (Mintz, 2013). Neufeld et al. structured their SBIRT course around live clinical observation and patient interviews (Neufeld et al., 2012). Muzyk et al. included supervised real-patient counseling sessions with feedback, using a validated assessment tool (BECCI) (Muzyk et al., 2019). Selzer et al. argued for grounding psychotherapy concepts in clinical realities, particularly via bedside teaching and the discussion of live cases (Selzer et al., 2015). King et al. added realism by integrating role play with clinical case simulations (King et al., 2015). Chéret used real-case narratives as a base for discussion (Chéret et al., 2018). Daeppen evaluated clinical exposure during addiction-focused clerkships, demonstrating increased student motivation (Daeppen et al., 2012). Dobkin et al. involved students in mindfulness-based clinical interventions and found gains in their relational skills (Dobkin & Hutchinson, 2013). Buck used narrative exposure from psychiatric in-patients to train empathic responses (Buck et al., 2021). These examples affirm that clinical exposition is important for contextualizing theory and fostering interpersonal understanding in psychotherapy education.

#### 3.2.4. Multimodal

Most effective programs adopted a multimodal approach, combining didactic sessions, experiential learning, reflection and clinical exposure. Mintz recommended multiple touchpoints for teaching psychodynamic principles, including lectures, case discussions and clinical integration (Mintz, 2013). Selzer et al. emphasized simplicity, clinical relevance and adaptability in combining theoretical, experiential and reflective teaching (Selzer et al., 2015). Neufeld et al. and Muzyk et al. used structured curricula that blended lectures, skills labs, patient interaction and interprofessional collaboration (Neufeld et al., 2012; Muzyk et al., 2019). Merlo et al. implemented a CBT-based session using video lectures, vignettes, group activities and role play, with excellent feedback (Merlo et al., 2022). Buck, Chéret, Edwards and Katlman all employed multiple teaching strategies (including narrative cases, peer reflection, digital tools and supervised practice), showing gains in student engagement and clinical readiness (Edwards et al., 2022; Kaltman et al., 2015; Chéret et al., 2018; Buck et al., 2021). Truong et al., in their systematic review, concluded that multimodal methods were associated with stronger learner outcomes, although few studies met rigorous standards (Truong et al., 2015). Even Keifenheim, McKenzie and Daeppen emphasized the combination of didactic, reflective and clinical components, indicating a trend toward layered learning strategies (Keifenheim et al., 2019; McKenzie et al., 2012; Daeppen et al., 2012). Multimodal formats best reflect the complexity of psychotherapy training by integrating cognitive, affective and behavioral domains.

### 3.3. Quality Appraisal

The BEME assessment revealed that most studies were of moderate-to-high methodological quality, with eight studies being rated as high quality and six being rated as moderate. Only one study was rated as low quality. The strongest aspects across studies were the clarity of objectives and the detailed description of the educational interventions. However, several studies showed limitations in the validity of outcome measures, data analysis transparency and, in some cases, sampling strategy. Among the quantitative studies assessed with the MERSQI, scores reflected moderate methodological appraisal, particularly in domains such as study design and outcome measurement, though external validity and instrument validation were often under-reported. These findings suggest a generally solid but heterogeneous evidence base, underscoring the need for more designed and transparently reported educational research in psychotherapy training. Table 2 represents the BEME scores, while Table 3 depicts the MERSQI assessments.

## 4. Discussion

This scoping review aimed to identify the best practices for teaching psychotherapy to medical students. A total of 15 studies were fully analyzed, revealing four main categories of teaching methods: e-learning, role play, clinical exposition and multimodal approaches. Multimodal strategies were the most frequently used and often combined lectures, clinical experiences, digital content and interactive exercises. Role play and clinical exposition were also widely implemented and valued for their ability to enhance communication skills, empathy and therapeutic understanding. E-learning was less common but offered flexibility and was often used in blended formats. The overall methodological quality of the included studies was moderate to high, based on structured evaluations using the BEME checklist and the MERSQI for quantitative studies. These findings support the integration of diverse, experience-based methods, particularly when combined, in the teaching of psychotherapy to future physicians.

In the field of medical education, various methodologies have been employed to teach psychotherapy to medical students, each with its own set of advantages and challenges. E-learning has emerged as a flexible and scalable approach in psychotherapy training. A systematic review by Mikkonen et al. evaluated e-learning programs in psychotherapy training, finding that such programs are generally associated with positive learning outcomes, including trainee satisfaction and knowledge acquisition (Mikkonen et al., 2024). The review also noted that e-learning methods often show equivalence to conventional training methods in terms of learning outcomes. However, the authors emphasized the need for further research to establish global standards for e-learning in psychotherapy education and to assess the impact on patient outcomes.

Role play is widely recognized for enhancing communication skills and empathy among medical students. Our review aligns with findings from a study by Garcia-Huidobro et al., which introduced a comprehensive therapy decision-making course incorporating various educational strategies, including application seminars that utilized role play techniques (Garcia-Huidobro et al., 2024). Students reported increased self-efficacy and perceived importance of various aspects of therapy decision-making, highlighting the effectiveness of interactive methods like role play in medical education.

Clinical exposition, involving direct patient interaction, is pivotal for contextualizing theoretical knowledge. This demonstrates that situational awareness is important to transition from theory to practice. Feller et al. demonstrated this concept in their narrative review, which aligns with the identified studies of this scoping review (Feller et al., 2023). This underscores the value of clinical exposure in mastering practical competencies that are essential for psychotherapy practice.

Multimodal approaches, which integrate various teaching methods such as lectures, role play and clinical exposure, have consistently been shown to enhance psychotherapy training. For instance, a protocol outlined by Pei et al. describes a randomized controlled trial evaluating the effectiveness of a 2-day intensive educational intervention for medical students and residents in China that combines didactic content with experiential components (Pei et al., 2025). This study reflects a growing international recognition that layered, interactive teaching strategies are essential for developing psychotherapeutic competencies. Such integrated formats not only improve knowledge acquisition but also foster empathy, self-reflection and clinical confidence, which are critical skills for patient-centered mental healthcare. As medical education evolves to meet complex psychosocial demands, multimodal approaches offer a scalable and evidence-informed path forward.

Beyond the limited inclusion of psychotherapy per se, the findings of this review reflect a deeper structural issue within medical education—the persistent privileging of biomedical interventions over psychosocial approaches. Many general physicians report comfort in prescribing psychotropic medications but hesitate to engage in psychotherapeutic conversations or techniques, not solely due to lack of technical training, but because such skills are often undervalued in their formative years. This discrepancy may stem from hidden curricula, time pressures or a perceived lack of legitimacy or ownership over psychotherapeutic care. It suggests a need not only for more content on psychotherapy, but for a cultural shift within medical education that reinforces the value of relational, empathic and psychological competencies as being a core skill in competent medical practice. Addressing this imbalance requires the deliberate integration of mental health literacy, reflective practice and psychotherapeutic principles throughout the curriculum, as well as institutional recognition of their relevance across all specialties.

It is important to highlight the limitations of this literature review. Although a comprehensive search strategy was employed across multiple databases, it is possible that relevant studies published in non-indexed journals or the gray literature were missed. While articles in both English and French were included, the exclusion of studies in other languages may have led to language bias. Also, the heterogeneity of study designs, populations and outcome measures made a direct comparison between studies difficult, limiting the ability to synthesize quantitative findings. Additionally, although a quality assessment was conducted using validated tools, the scoring remains somewhat subjective and may vary depending on interpretation. Because scoping reviews aim to map the breadth of available evidence rather than evaluate effectiveness, no conclusions can be drawn regarding the superiority of one teaching method over another. It is also important to note that most included studies assessed outcomes immediately post-intervention, limiting the ability to determine the sustainability of changes over time.

The findings of this review have implications for curriculum development in undergraduate medical education. By demonstrating the effectiveness of multimodal strategies (particularly those that incorporate active learning techniques such as role play and clinical immersion), this review supports the integration of structured psychotherapy training into core medical curricula rather than limiting it to optional electives. Educators and curriculum designers can draw on these insights to embed psychotherapeutic content longitudinally, aligning it with important developmental stages of medical training and reinforcing competencies in communication, empathy and patient-centered care. Moreover, incorporating brief, evidence-based modalities like motivational interviewing or cognitive restructuring into clinical rotations can enhance relevance and feasibility, especially in time-constrained programs. Interprofessional learning environments and blended teaching formats, including e-learning, can further increase accessibility and scalability.

To effectively implement psychotherapy training within diverse institutional contexts, medical educators should adopt scalable and adaptable strategies. For programs with limited resources, incorporating brief, low-cost interventions such as structured role play sessions during existing communication skills modules or clinical rotations can offer high-impact learning opportunities. Where faculty expertise is limited, digital resources (including recorded demonstrations, e-learning modules or online case simulations) can supplement in-person instruction. Institutions with more extensive resources may consider developing longitudinal psychotherapy tracks or elective immersion programs that integrate didactic content with supervised clinical experiences. Collaborating with departments of psychiatry, psychology or social work can also facilitate interprofessional teaching and reduce faculty burden. Regardless of setting, aligning psychotherapy training with core competencies (such as empathy, therapeutic communication and reflective practice) ensures its relevance across specialties and supports sustainability within broader medical education frameworks.

## 5. Conclusions

This scoping review highlights the diverse and evolving landscape of psychotherapy education for medical students, identifying four primary teaching modalities: e-learning, role play, clinical exposition and multimodal strategies. Among these, multimodal approaches emerged as the most frequently employed and pedagogically effective. Role playing and clinical immersion were particularly valuable for cultivating empathy, communication skills and therapeutic insight, while e-learning enhanced accessibility and flexibility, especially when paired with interactive formats. Although the overall quality of the included studies was moderate to high, heterogeneity in design and outcome reporting underscores the need for more methodologically rigorous and longitudinal research in this field. These findings suggest that to meaningfully improve psychotherapy training in undergraduate medical education, programs should prioritize active learning methods and embed psychotherapeutic competencies into required curricula rather than relegating them to optional electives. Faculty can leverage brief, structured interventions (such as motivational interviewing workshops or simulated patient encounters) to teach core communication and relational skills. For institutions with limited resources, blended-learning formats combining online modules with small-group discussions offer a scalable solution. Collaborations with psychiatry, psychology or social work departments can support interprofessional teaching and diversify instructional approaches. Overall, enhancing psychotherapy education equips future physicians to deliver more holistic, patient-centered care and strengthens their ability to address the complex psychosocial needs of diverse patient populations. Medical schools should view the integration of psychotherapy not as an optional enrichment, but as a foundational element of comprehensive medical training.

## Figures and Tables

**Table 1:** Summary of the identified studies.

Authors Year	Title	Population	Type of Psychotherapy	Teaching Methods	Results
Buck et al. (2021)	The physician healer track: educating the hearts and the minds of future physicians	Medical students; n = 258	Training in mindfulness, cognitive–behavioral therapy, nonviolent communication, motivational interviewing, spirituality in healthcare, wellness, equanimity and ‘being with suffering’ is reinforced across all 4 years. Community building and reflection are integral to the training both in the monthly sessions and the immersion courses.	The physician healer track—a 500-contact-hour curricula integrated over 4 years; monthly dinner meetings with faculty mentors; a two-month preceptorship in the first year; a one-month immersion course in MS4 and one elective.	Less than 1% attrition. In serial assessments, students report continued growth in personal development, professional development and the ability to empathize. Enrollment has grown from 26 students in the first year (11% of class) to a total of 258 students across our first 6 years (average of 20–26% of each class).
Chéret et al. (2018)	Motivational interviewing training for medical students: a pilot pre-post feasibility study	Medical students; n = 20	Motivational interviewing.	Three four-hour sessions of MI training over one week; viewing and commenting on video clips; practical exercise; lectures and the distribution of memory aids; role playing.	Global scores from the MITI-3.1.1 code, including “MI-Spirit”, were attributed to the audiotaped interviews by two independent coders, who were blinded to the pre- or post-training status of the interview. Secondary outcomes were caregivers’ perception of students’ empathy (CARE questionnaire), students’ evaluation of self-efficacy to engage in a patient-centered relationship (SEPCQ score) and students’ satisfaction with their own performance (analog scale). MI-Spirit score increased significantly after training (*p* < 0.0001; effect size 1.5). Limited improvements in CARE score (*p* = 0.034; effect size 0.5) and one of the SEPCQ dimensions (sharing information and power with the patient; *p* = 0.047; effect size 0.5) were also noted. Students’ satisfaction score was unaffected (*p* = 0.69). These findings suggest that brief MI training can improve communication skills in medical students.
Daeppen et al. (2012)	Training medical students to conduct motivational interviewing: a randomized controlled trial	All students (n = 131) in year five of a six-year curriculum at Lausanne University Medical School in Switzerland were randomized into an experimental group (n = 66) or control group (n = 65)	Motivational interviewing.	Four-hour sessions of practical exercises and role playing. After training all students in basic communication skills in years two and three (control condition), an eight-hour MI training workshop was completed by students in the experimental group. One week after the training, students in both groups were invited to meet for 15 min with two standardized patients.	Motivational interviewing (MI) skills were coded by four blinded research assistants using the Motivational Interviewing Treatment Integrity (MITI) coding system, version 3.0. A superior performance was shown among trained versus control students, as demonstrated by higher mean values for empathy and MI spirit. Behavior- counts assessment demonstrated better performance in MI among trained versus control students regarding occurrences of MI-adherent behavior, MI nonadherent behavior, closed questions, open questions, simple reflections and complex reflections. In sum, an eight-hour training workshop was associated with improved MI performance, lending support for the implementation of MI training in medical schools.
Dobkin and Hutchinson (2013)	Teaching mindfulness in medical school: where are we now and where are we going?	Medical students (literature review on teaching mindfulness)	Mindfulness.	A wide range of formats are used in teaching mindfulness. These include simple lectures, 1-day workshops and 8–10 week programs in mindfulness-based stress; an 8-week course delivered in sessions of 2.5 h each week, with a silent retreat day; and lectures or workshops on mindfulness are interspersed throughout the 4-year curriculum.	Outcomes identified thus far include reductions in perceived stress, anxiety and symptoms of depression, as well as increases in mindfulness, empathy and self- compassion, as measured soon after the course. We have yet to learn if these changes are maintained over time. Moreover, we do not know how these impacts generalize to other aspects of the students’ lives and their work with peers, colleagues and patients.
Edwards et al. (2022)	Teaching brief motivational interviewing to medical students using a pedagogical framework	Pre-clinical medical students; n = 46	Motivational interviewing.	Educational intervention premised on the *Learn, See, Practice, Prove, Do, Maintain* pedagogical framework, comprising 2 × 2 h lectures, a 2 h role play triad session and 3 × 2 h small-group simulated patient encounters supported by scaffolding strategies.	Students who received brief MI training improved in knowledge and confidence from baseline to post-training; gains remained at 3 months. Brief MI skills improved across the simulation sessions.
Kaltman et al. (2015)	Enhancing motivational interviewing training in a family medicine clerkship	3rd year medical students; n = 16	Motivational interviewing.	An online learning community for enhanced training in motivational interviewing was developed for 3rd-year medical students. The website included educational materials about motivational interviewing, as well as problematic health behaviors, a repository of exemplar videos and student videos with feedback and a discussion board. Student participants were given the opportunity to record an encounter with a patient and to receive feedback on their use of motivational interviewing from a faculty member.	Student volunteers in the Family Medicine Clerkship at Georgetown University School of Medicine were randomized to enhanced training, which included the online learning community or training as usual. Students in the enhanced training arm were rated as having significantly higher scores in motivational interviewing style in the observed structured clinical exam than training-as-usual students.
Keifenheim et al. (2019)	“A change would do you good”: Training medical students in Motivational Interviewing using a blended-learning approach—A pilot evaluation	A mandatory MI course was implemented for sixth-semester medical students	Motivational interviewing.	A mandatory MI course/e-learning/blended learning is defined as a combination of e-learning and classical teaching formats, such as lectures and small groups.	This pilot study suggests that basic MI skills can be successfully taught in a blended-learning teaching approach. Further research should investigate sustainability and transfer to clinical practice.
King et al. (2015)	All the world’s a stage: evaluating psychiatry role-play based learning for medical students	A total of 107 students from three clinical schools of the University of Melbourne	All types (communication skills).	Role play-based learning (RBL), as a vehicle for teaching psychiatry.	Respondents were overwhelmingly positive about the sessions, reporting benefits for learning, communication skills, insight and confidence in managing both assessment tasks and real patients.
McKenzie et al. (2012)	Medical and psychology students’ knowledge of and attitudes towards mindfulness as a clinical intervention	A total of 91 medical students from Monash University, 49 medical students from Deakin University and 31 psychology students from Deakin University	Mindfulness.	All types of teaching compared to no teaching.	Medical students with exposure to mindfulness in their course have a greater knowledge of it and are more likely to administer it or recommend it compared to medical students without exposure. Knowledge of mindfulness is positively correlated with students’ willingness to use or recommend it. The inclusion of mindfulness exposure in medical courses and possibly also in psychology courses may help mindfulness fulfill its clinical potential and increasingly benefit patients who are suffering from a range of clinical conditions.
Merlo et al. (2022)	Introduction to Cognitive Restructuring for Medical Students	A total of 139 first-year medical students and 152 second-year medical students	Cognitve–behavioral therapy.	A 90-min learning activity, which included a short videotaped lecture, clinical case vignette, small-group discussion and application exercise.	Immediately following the learning activity, students and faculty completed anonymous evaluations. For the first-year cohort, upwards of 80% of students and 100% of faculty respondents rated the session either good or excellent. For the second-year cohort, over 80% of students and over 90% of faculty rated the session as either good or excellent. Approximately 90% of first- and second-year medical students and 100% of faculty recommended offering the session to future students. Open-ended feedback from students was overwhelmingly positive.
Mintz (2013)	Teaching psychoanalytic concepts, skills and attitudes to medical students	Medical students	Psychodynamic.	Multimodal approaches.	The authors argue that teaching psychoanalytic concepts to medical students (such as empathy, transference and the doctor–patient relationship) remains valuable despite their decline in curricula. They suggest practical ways to integrate these ideas through lectures, clinical experiences and electives. While challenges like limited resources and student resistance exist, incorporating psychodynamic perspectives can enhance professionalism, improve patient care and support recruitment into psychiatry.
Muzyk et al. (2019)	Behavior change counseling of patients with substance use disorders by health professions students	A total of 78 students from medicine, nursing, pharmacy, social work and physician assistant programs completed the one-month course, with 53 of them counseling a patient	Motivational interviewing.	Each course included four in-person classes with a total of six hours of instructional time. Class topics focused on (1) developing empathy and examining personal bias; (2) the behavioral change counseling model and implications for its use in healthcare settings; and (3) the recognition, screening and treatment of SUDs. Following these class sessions, students had an opportunity to counsel a patient with an SUD in the clinical setting using behavioral change counseling and receive observation, assessment and feedback from a faculty member experienced in this technique.	Students’ BECCI-rated counseling skills indicated that they performed the recommended counseling practices and spoke for “less than half the time” or for “about half the time” when counseling. A total of 93% of SUD patients reported a willingness for follow-up care about their substance use after the student-led session.
Neufeld et al. (2012)	A collaborative approach to teaching medical students how to screen, intervene and treat substance use disorders	Second- year medical students; n = 118	Motivational interviewing.	A collaborative 15-h course using the SBIRT (Screening, Brief Intervention and Referral to Treatment) model to teach second-year medical students about substance use disorders. The 15-h course (39 faculty teaching hours) arose from a collaboration between faculties in the Departments of Medicine and Psychiatry and included 5 h of direct patient interaction during clinical demonstrations and in small-group skills development.	The course significantly improved students’ knowledge, as shown by a marked increase in post-test scores. Key features included interactive patient sessions, small-group skills training and interdepartmental collaboration between psychiatry and internal medicine. The course was well received and demonstrated the feasibility and effectiveness of experiential, integrated teaching on addiction in medical education.
Selzer et al. (2015)	Teaching psychological processes and psychotherapy to medical students	Medical students	All types.	Having a familiar information scaffold, as well as associating the teaching with a patient, makes the learning memorable, personal and relevant. Demonstrating this eclecticism shows that we can be flexible and suit the theory and practice to patient needs, that is, avoiding blind allegiance to one theory or another.	Teaching psychotherapy and psychological processes to medical students need not be difficult. We have described some of the techniques we have used successfully.
Truong et al. (2015)	What Is the Efficacy of Teaching Psychotherapy to Psychiatry Residents and Medical Students?	Medical students and residents	All types.	Multimodal approaches.	There is a need for more systematic evaluations and publications relating to psychotherapy teaching practices for both medical students and residents. Researchers are encouraged to study educational approaches across these groups, while training and clerkship directors should be surveyed on their content, methods and outcomes.

**Table 2:** BEME assessments.

Article	BEME Score (0–11)	BEME Rating
Buck et al. (2021)	8	Moderate
Chéret et al. (2018)	7.5	Moderate
Daeppen et al. (2012)	7	Moderate
Dobkin and Hutchinson (2013)	6.5	Low
Edwards et al. (2022)	8.5	Moderate
Kaltman et al. (2015)	8	Moderate
Keifenheim et al. (2019)	9	High
King et al. (2015)	8	Moderate
McKenzie et al. (2012)	6	Low
Merlo et al. (2022)	9	High
Mintz (2013)	7.5	Moderate
Muzyk et al. (2019)	10	High
Neufeld et al. (2012)	9.5	High
Selzer et al. (2015)	8.5	Moderate
Truong et al. (2015)	10.5	High

**Table 3:** MERSQI assessments.

Article	Study Design (0–3)	Sampling (0–3)	Type of Data (0–3)	Validity (0–3)	Analysis (0–3)	Outcomes (0–3)	Total MERSQI (0–18)	MERSQI Rating
Edwards et al. (2022)	2	2	2	1	2	2	11	Low
Keifenheim et al. (2019)	2	2	2	2	2	2	12	Moderate
King et al. (2015)	2	2	2	1	2	2	11	Low
Muzyk et al. (2019)	2	2	2	2	3	3	14	Moderate
Neufeld et al. (2012)	2	2	2	1	2	2	11	Low
Truong et al. (2015)	3	3	2	2	3	3	16	High

## Data Availability

Not applicable.

## Supplementary Materials

The following supporting information can be downloaded at https://www.mdpi.com/article/10.3390/bs15060780/s1 (Tricco et al., 2018).

## Abbreviations

The following abbreviations are used in this manuscript:

BEME	Best Evidence Medical Education
CBT	Cognitive–behavioral therapy
MD	Doctor of Medicine
MERSQI	Medical Education Research Study Quality Instrument
MI	Motivational interviewing
MITI	Motivational Interviewing Treatment Integrity
PRISMA-ScR	Preferred Reporting Items for Systematic Reviews and Meta-Analyses Extension for Scoping Reviews
SBIRT	Screening, Brief Intervention and Referral to Treatment
