# Supplementary material for: Best Practices for Teaching Psychotherapy to Medical Students: A Scoping Review
Source: Behav Sci (Basel). 2025 Jun 5;15(6):780. doi: 10.3390/bs15060780 (PMC12189545; doi:10.3390/bs15060780)
Supplement: Supplementary file 1 [file behavsci-15-00780-s001.zip › TableS1.pdf]

Table S1. Teaching Psychotherapy – Medical Students

Concept Map

**Medical students**	**Psychotherapy**	**Teaching**
**Descriptors (Mesh)**<br><br>"Students, Medical"[Mesh]<br>"Clinical Clerkship"[Mesh]<br><br><br>**Keywords (title)**<br><br>Internship(s)<br>Intern(s)<br>Clerkship(s)<br>Medical student(s)<br>Medical school(s)	**Descriptors (MeSH)**<br><br>"Psychotherapy"[Mesh]<br>“Cognitive Behavioral Therapy” [MeSH]<br>“Psychotherapy, Psychodynamic” [MeSH]<br>“Defense Mechanisms” [MeSH]<br>“Transference, Psychology” [MeSH]<br><br>**Keywords (title)**<br><br>Psychotherapy(ies)<br>Psychotherapeutic(s)<br>Support therapy<br>Cognitive behavioral therapy<br>Cognitive behavioural therapy<br>Psychodynamic therapy<br>Defense mechanisms<br>Transference<br>Countertransference	**Descriptors (MeSH)**<br><br>"Teaching"[Mesh]<br>"Learning"[Mesh]<br>"Education, Medical"[Mesh]"<br><br><br>**Keywords (title)**<br><br>Medical education<br>Teaching<br>Teach<br>Learn<br>Learning<br>Training<br>Train<br>Curriculum(s)

Search strategy:

("Students, Medical"[Mesh] OR "Clinical Clerkship"[Mesh] OR Internship[TITLE] OR internships[TITLE] OR Intern[TITLE] OR interns[TITLE] OR Clerkship[TITLE] OR clerkships[TITLE] OR "Medical student"[TITLE] OR "medical students"[TITLE] OR "medical school"[TITLE] OR "medical schools"[TITLE]) **AND** ("Psychotherapy"[Mesh] OR "Cognitive Behavioral Therapy"[MeSH] OR "Psychotherapy, Psychodynamic" [MeSH] OR "Defense Mechanisms"[MeSH] OR "Transference, Psychology" [MeSH] OR Psychotherapy[TITLE] OR psychotherapies[TITLE] OR Psychotherapeutic[TITLE] OR psychotherapeutics[TITLE] OR "support therapy"[TITLE] OR "cognitive behavioral therapy"[TITLE] OR "cognitive behavioural therapy"[TITLE] OR "psychodynamic therapy"[TITLE] OR "Defense mechanisms"[TITLE] OR "Transference"[TITLE] OR "Countertransference"[TITLE]) **AND** ("Teaching"[Mesh] OR "Learning"[Mesh] OR "Education,

Medical"[Mesh] OR Teaching[TITLE] OR teach[TITLE] OR Learning[TITLE] OR learn[TITLE] OR training[TITLE] OR train[TITLE] OR course[TITLE] OR courses[TITLE] OR curriculum[TITLE] OR curriculums[TITLE] OR "Medical education"[TITLE])
